# Clinical Validation of Targeted and Untargeted Metabolomics Testing for Genetic Disorders: A 3 Year Comparative Study

**DOI:** 10.1038/s41598-020-66401-2

**Published:** 2020-06-10

**Authors:** Naif A. M. Almontashiri, Li Zha, Kim Young, Terence Law, Mark D. Kellogg, Olaf A. Bodamer, Roy W. A. Peake

**Affiliations:** 1 Department of Laboratory Medicine, Boston Children’s Hospital, Harvard Medical School, Boston, Massachusetts USA; 2 Faculty of Applied Medical Sciences and the Center for Genetics and Inherited Disorders, Taibah University, Almadinah Almunwarah, Saudi Arabia; 3 Division of Genetics and Genomics, Boston Children’s Hospital, Harvard Medical School, Boston, Massachusetts USA; 4 Broad Institute of Harvard University and MIT, Cambridge, Massachusetts USA

**Keywords:** Biochemistry, Metabolomics, Biomarkers, Diagnostic markers

## Abstract

Global untargeted metabolomics (GUM) has entered clinical diagnostics for genetic disorders. We compared the clinical utility of GUM with traditional targeted metabolomics (TM) as a screening tool in patients with established genetic disorders and determined the scope of GUM as a discovery tool in patients with no diagnosis under investigation. We compared TM and GUM data in 226 patients. The first cohort (n = 87) included patients with confirmed inborn errors of metabolism (IEM) and genetic syndromes; the second cohort (n = 139) included patients without diagnosis who were undergoing evaluation for a genetic disorder. In patients with known disorders (n = 87), GUM performed with a sensitivity of 86% (95% CI: 78–91) compared with TM for the detection of 51 diagnostic metabolites. The diagnostic yield of GUM in patients under evaluation with no established diagnosis (n = 139) was 0.7%. GUM successfully detected the majority of diagnostic compounds associated with known IEMs. The diagnostic yield of both targeted and untargeted metabolomics studies is low when assessing patients with non-specific, neurological phenotypes. GUM shows promise as a validation tool for variants of unknown significance in candidate genes in patients with non-specific phenotypes.

## Introduction

Inborn errors of metabolism (IEMs) represent a large group of genetic disorders caused by deficiencies in proteins of intermediary metabolism among others, leading to accumulation of toxic compounds, and/or abnormal energy metabolism. The traditional paradigm for identification of IEMs involves hypothesis-driven phenotyping and/or newborn screening followed by targeted assays to determine the biochemical phenotype^1^. This process often concludes with molecular testing for identification of causative variants. Although this approach is highly effective for the majority of known IEMs, there are phenotypes for which the genetic and metabolic basis of disease remains to be discovered. Next generation sequencing, including whole exome sequencing (WES), has emerged as the most important tool for discovery of novel IEMs and for conditions exhibiting a broad phenotypic spectrum^2^. Despite the scope of WES, a large proportion of investigations do not yield a genetic diagnosis, often resulting in the identification of variants of unknown significance (VUS) in either disease-associated or candidate genes^3^. As such, the concept of “functional genomics” has evolved to determine the functional characteristics of VUS^4^.

Metabolomics is defined as the comprehensive analysis of metabolites within a biological system^5^. Metabolomics studies have been used for the investigation of IEMs and is casually classified into two categories; targeted and global, untargeted metabolomics. In targeted metabolomics (TM), pre-selected, specific compounds are analyzed and compared with age dependent reference ranges. This approach involves the measurement of pre-defined, chemically-characterized analytes, such as organic acids, amino acids and acylcarnitine species^6^. TM analytics require selective sample preparation steps optimized for the physical-chemical properties of the compounds of interest, followed by analysis using various types of chromatography and/or mass spectrometry. The drawback of this approach is the requirement for an *a priori* hypothesis, the use of sequential assays and lastly, the lack of scope for integration with multi-omics, such as WES^7^.

Global untargeted metabolomics (GUM) aims to identify a maximum number of metabolites in a single sample, generating a metabolomic fingerprint, ideally characteristic for a particular biochemical phenotype. This is achieved through the use of platforms consisting of multiple, high-specification synchronized chromatography-mass spectrometry systems with powerful informatics pipelines^8^. Thus, GUM has potential for biomarker discovery and functional characterization of VUS identified through whole exome and genome sequencing^9,10^. For the study of IEM, TM is already well established and validated in biochemical genetics laboratories; in contrast, GUM platforms have only recently entered the realm of clinical diagnostics^11,12^. As GUM transitions into the clinical diagnostics arena, it has been suggested that it may replace conventional targeted approaches^13^. However, prior to its integration into a clinical diagnostics workflow, a comprehensive evaluation of their analytical and clinical utility is required.

We evaluated the clinical utility of a commercially available GUM platform through comparison with conventional TM assays in two distinct patient cohorts. The first cohort comprised patients with confirmed biochemical and/or molecular diagnoses. The second cohort comprised individuals without confirmed diagnosis who were under ongoing diagnostic evaluation at the time of the study. Therefore, the objectives of this study were twofold: (i) to determine the clinical utility of GUM in the evaluation of patients with known IEM and other genetic syndromes; (ii) to assess the performance of GUM as a discovery tool in patients under investigation for an IEM or genetic disorder with no established diagnosis.

## Results

### Metabolite study

A full description of the metabolite comparison study is summarized in the supplementary materials (Table S1). Concordance between targeted and untargeted analyses was defined as the number of specimens in which both approaches detected an increase or decrease of a given metabolite expressed as a percentage of the total number of samples compared for that metabolite. The number of patient samples available for comparison for a given metabolite ranged from 1 to 22. The mean level of concordance was 50% (range: 0–100%) across 81 metabolites compared using both approaches.

### Clinical utility study

A complete description of the diagnostic cohort is provided (Supplemental Table S2). A total of 51 clinically-relevant metabolites analyzed using both TIM and GUM were available for comparison. The ability of GUM to detect the 51 diagnostic metabolites yielded a diagnostic sensitivity of 86% (95% CI: 78–91) against TM (Fig. 2a). The data was further examined for potentially clinically-relevant discrepancies between the two approaches for the groups of disorders studied (Fig. 2b).

**Figure 1:**
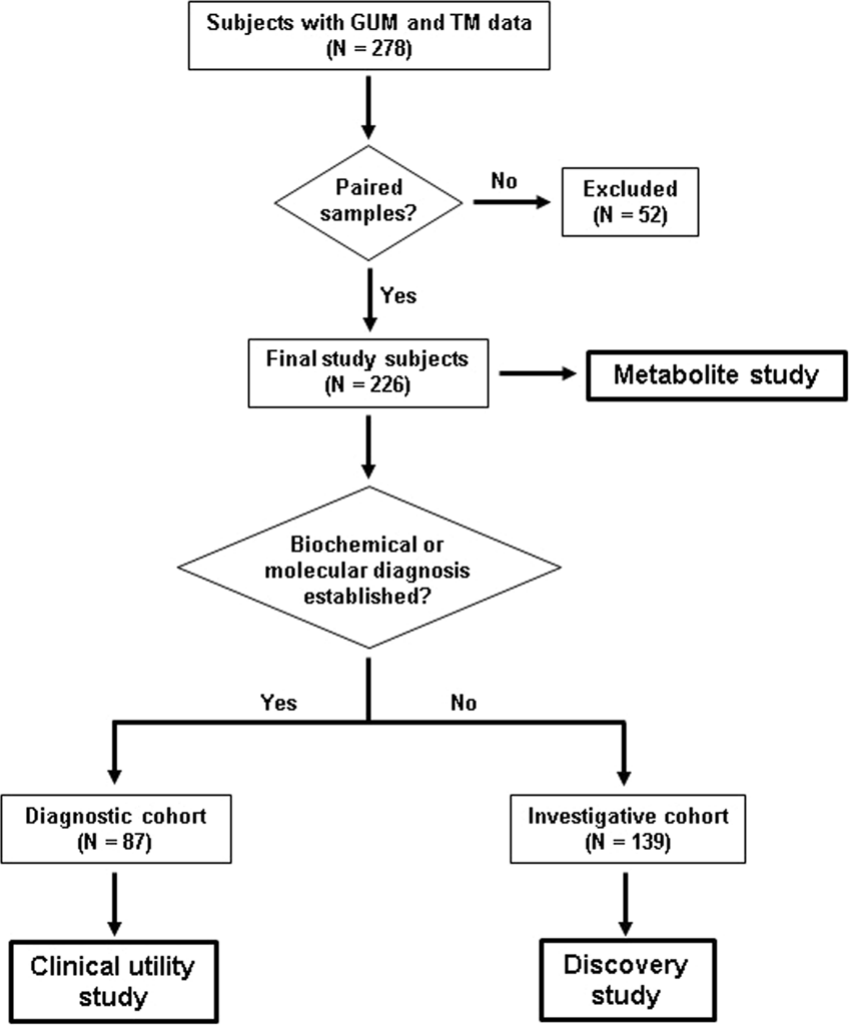
Flowchart describing patient selection for study. Patient records were interrogated in which GUM and TM studies were performed on paired samples. Data was compared in patients with established diagnoses (clinical utility study) and in patients with no established diagnosis (discovery study). The level of concordance between GUM and TM were compared in all samples (metabolite study).

**Figure 2:**
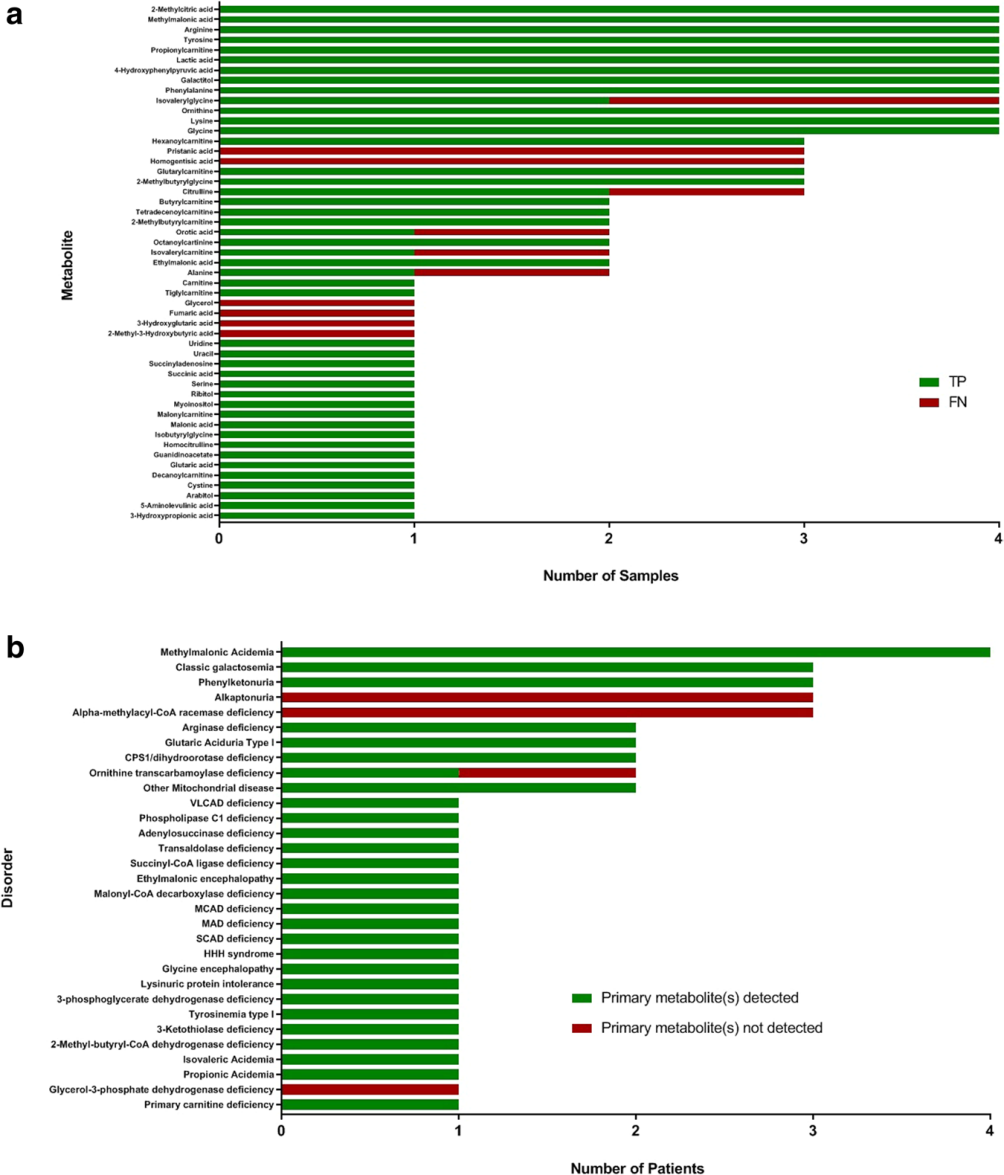
Diagnostic performance of GUM in the clinical utility study. GUM was compared with TM for, (**a**) the ability to detect 51 diagnostic metabolites in specimens collected from 87 patients with established IEMs or genetic disorders. Y-axis represents diagnostically-relevant metabolites, and x-axis represents the number of samples (plasma, urine and CSF) in which metabolite was detected. Metabolite data was expressed as a function of true positives (TP) and false negatives (FN); (**b**) the ability to successfully identify patients with disorders based on the detection of the primary metabolite associated with that disorder. Disorders are presented on the y-axis, and the number of patients detected with each disorder represented on the x-axis.

#### Organic acid disorders

In patients with propionic and methylmalonic acidemias, all key metabolites of propionyl-CoA were detected by GUM analysis. Although GUM failed to detect isovalerylglycine (IVG) in a sample collected from a patient with isovaleric acidemia (Table S2: Case F6), additional metabolites were detected that would have led to the correct diagnosis. A similar situation was apparent in a patient with glutaric aciduria type I (Table S2: Case F7), whereby GUM failed to detect plasma 3-hydroxyglutaric acid, present at 370 ng/mL (reference interval: 0–64) using TM. However, both approaches successfully identified elevations in glutarylcarnitine and glutaric acid. In contrast, GUM analysis of urine collected from 3 individuals from the same family with alkaptonuria (Table S2: Family F11) failed to detect homogentisic acid.

#### Disorders of amino acid metabolism, synthesis and transport

All diagnostically-relevant metabolites associated with phenylketonuria, tyrosinemia type I, non-ketotic hyperglycinemia, cystinuria and lysinuric protein intolerance were successfully detected using both TM and GUM. Patients with 3-phosphoglycerate dehydrogenase deficiency showed concurrent results for plasma serine by both methods (Table S2: Cases F17, F18). Additionally, GUM revealed low levels of plasma sphingomyelin in these patients, a potential biomarker for the disorder^16^.

#### Disorders of the urea cycle and related diseases

Eight patients with defects of the urea cycle and related disorders of nitrogen elimination were included. In one female carrier of ornithine transcarbamoylase (OTC) deficiency (Table S2: Case F23), urine orotic acid was mildly increased at 2.1 mmol/mol creatinine (reference interval: <0.1) by TM, but was not detected by GUM. Plasma levels of pyrimidine metabolites, recently proposed as potential biomarkers in OTC deficiency, were also detected using GUM analysis in this patient^17^. TM analysis of plasma from two patients with arginase deficiency (Table S2: Cases F20, F25) showed increases in guanidinoacetate with reciprocal decreases in ornithine using both approaches. In addition, GUM analysis of plasma revealed increases in several additional guanidino compounds, including homoarginine, N-acetylarginine and guanidinobutanoate, in agreement with a recent study^17^.

#### Disorders of fatty acid oxidation and energy metabolism

Comparative results were obtained in patients with short, medium, very long-chain and multiple acyl-CoA dehydrogenase deficiencies, and malonyl-CoA decarboxylase deficiency. Several patients with mitochondrial dysfunction were also studied. Urine from a patient with succinyl-CoA ligase deficiency (Table S2: Case F42) showed characteristic increases in adipic, lactic and succinic acids. There were four individuals included with molecular-confirmed mitochondrial disease; the presence of lactic acid was confirmed by both approaches in urine and plasma. Pyruvic acid levels were not formally assessed due to the challenges associated with stability of this analyte in blood^18^. Glycerol was not detected in the urine of a patient (Table S2: Case F40) with glycerol-3-phosphate dehydrogenase deficiency by GUM, detected using conventional targeted organic acid analysis.

#### Additional disorders

Apparent discrepancies were observed in data generated from three related patients with alpha-methylacyl-CoA racemase deficiency (Table S2: Family F44) where analysis of plasma using TM approaches demonstrated increased pristanic acid levels, not detected using GUM.

### Discovery study

**Figure 3:**
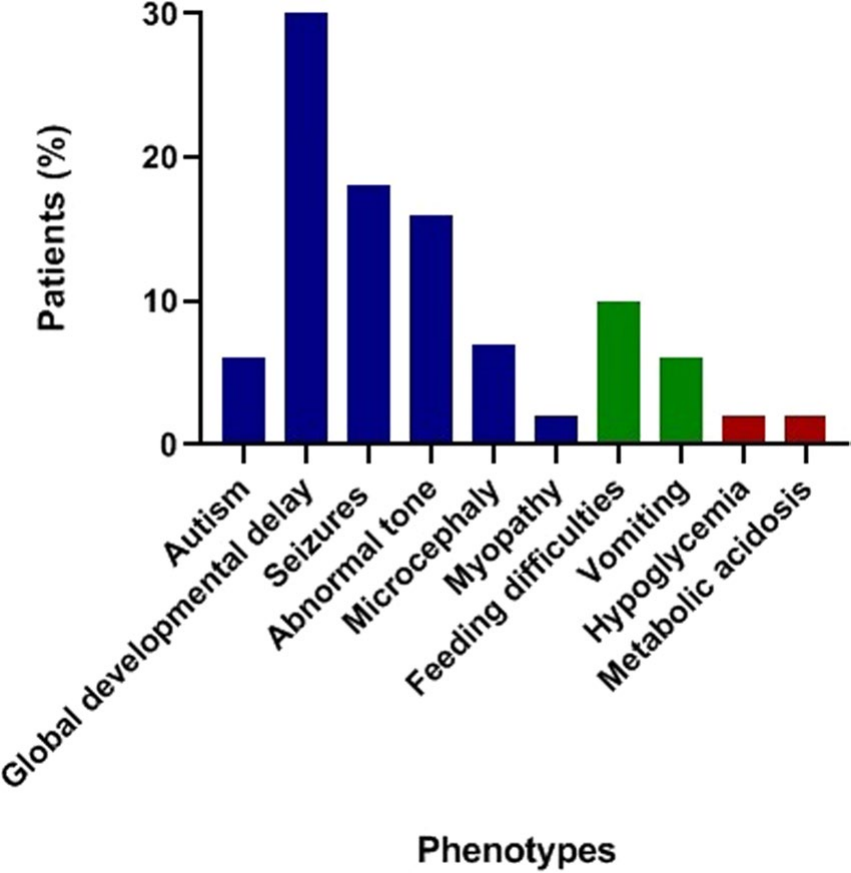
Breakdown of patients included in the discovery study. A summary of major phenotypes represented in the investigative cohort for the discovery study. Neurometabolic phenotypes are displayed in blue; gastrointestinal phenotypes are displayed in green; biochemical phenotypes are displayed in red.

The utility of GUM as a discovery tool was determined for the investigative cohort (n = 139). A summary of the cohort is described (Supplemental Table S3). A breakdown of patient phenotypes included in the study is shown in Fig. 3. Two thirds of patients had some neuro-developmental delay, epilepsy or abnormalities in muscle tone. 42 patients (30%) had apparently normal findings using both TM and GUM in plasma, urine or CSF. The remainder showed only mild perturbations in comparative metabolites using either approach. Of 177 specimens collected from 139 patients, a 7 year old male (Table S3: Case F162) with global developmental delay, epilepsy and skin/hair abnormalities was identified in which GUM analysis detected compounds related to the phenotype and/or genotype. WES revealed *de novo* heterozygous gain-of-function VUS in the *ODC1* gene. Extensive TM analysis conducted was unremarkable. In contrast, GUM analysis revealed increased levels of plasma N-acetylputrescine, an acylated form of putrescine. The *ODC1* gene, which encodes ornithine decarboxylase, is responsible for the conversion of ornithine to putrescine within the spermine synthetic pathway. The variant causes truncation of *ODC1* leading to the production of a mutant gene product with reduced degradation compared to wild-type, resulting in gain-of-function. This hypothesis was supported by GUM findings demonstrated by elevations in N-acetylputrescine, a novel biomarker for *ODC1* deficiency^19^.

## Discussion

We report, to the best of our knowledge, the findings of the first study comparing an untargeted clinical metabolomics platform with traditional targeted analyses for investigation of IEMs. We have adopted an unbiased approach through the use of sample pairing, and incorporated a diverse study population of genetic disorders. Through the provision of extensive metabolomic testing, this study has also contributed to the functional validation of gene variants across a large number of disorders.

The clinical utility study assessed the level of agreement between approaches for the detection of abnormal levels of diagnostic metabolite concentrations. Not surprisingly, concordance was found to be greatest in samples from confirmed IEM patients exhibiting profound perturbations in metabolite concentrations. GUM exhibited an estimated sensitivity of 86% for the 51 metabolites studied. Therefore, it may be inferred that the GUM platform would have led to the diagnosis in the vast majority of these cases. Perhaps the most obvious advantage of global metabolite assessment is the ability to detect multiple compounds in a holistic approach using a single analysis. As such, failure to detect a primary metabolite may be circumvented through detection of secondary metabolite(s). Additionally, many of these secondary metabolites are not covered by traditional TM assays, and may serve as novel biomarkers. However, in a small number of cases, failure to detect a primary metabolite may have resulted in a false negative result using GUM, as was the case of homogentisic acid (HGA) in alkaptonuria. HGA, a dihydroxyphenylacetic acid, is readily excreted in the urine of alkaptonuria patients, enabling easy detection using conventional organic acid analysis^20^. Similarly, failure to detect elevated pristanic acid levels in alpha-methylacyl-CoA racemase deficiency may have resulted in a false negative result^21^. It may also be inferred from one or two cases that targeted analysis was superior in detecting minor metabolite perturbations; namely, the detection of trace quantities of glycerol in the urine of a patient with glycerol-3-phosphate dehydrogenase deficiency using targeted organic acid analysis, or detection of urine orotic acid in a female carrier of OTC deficiency. There is also an advantage in having quantitative data with TM assays. Borderline low concentrations may not be appropriate under certain clinical conditions. In a case of asparagine synthetase deficiency, TM detected a low/normal asparagine concentration in CSF. This could be considered a significant finding within the clinical context and may lead to follow-up molecular studies. There is also a limitation in comparability of *z*-scores within an individual. In lieu of having clinical information, the relationship between metabolite levels is often suggestive of a diagnosis. For example, the abundance of urine malonic acid with respect to methylmalonic acid is informative for differential diagnosis of malonic aciduria. Although GUM successfully detected both analytes in our patient with malonyl-CoA decarboxylase deficiency, there was insufficient quantitative information to distinguish the disorder from combined malonic and methylmalonic aciduria (OMIM# 614265)^22^. Such observations are perhaps not unexpected. The ability of contemporary GUM platforms to detect multiple metabolites with such disparate physicochemical properties across wide concentration ranges in a semi-quantitative fashion is a noteworthy achievement^7,23^. However, some legitimate concerns remain as to whether they are truly ready to replace traditional TM assays as a first-line screening tool for IEMs.

A large number of IEMs remain unrecognized since their characteristic biomarkers are not measured by targeted assays, or the genetic basis/phenotype remains to be discovered. In our investigative cohort, routine targeted metabolomic work-up of 177 specimens collected from 139 patients provided no additional diagnostic information which could explain the phenotype and/or molecular findings. In contrast, GUM analysis in the same cohort successfully identified a case where a diagnostically-relevant metabolite was linked to the phenotype and genotype. N-acetylputrescine, an intermediate in the spermine synthetic pathway, was detected in a patient with a variant in *ODC1*^24^. Although this translates to a diagnostic yield of 0.7%, lower than observations from previous studies (estimated at 1–5% in patients with neuro-developmental symptoms), it does showcase the utility of GUM for validation of omics discoveries^25–30^. However, our data would also suggest that metabolomic testing, regardless of approach, is perhaps not highly conducive in patients with non-specific, neurological symptoms.

**Figure 4:**
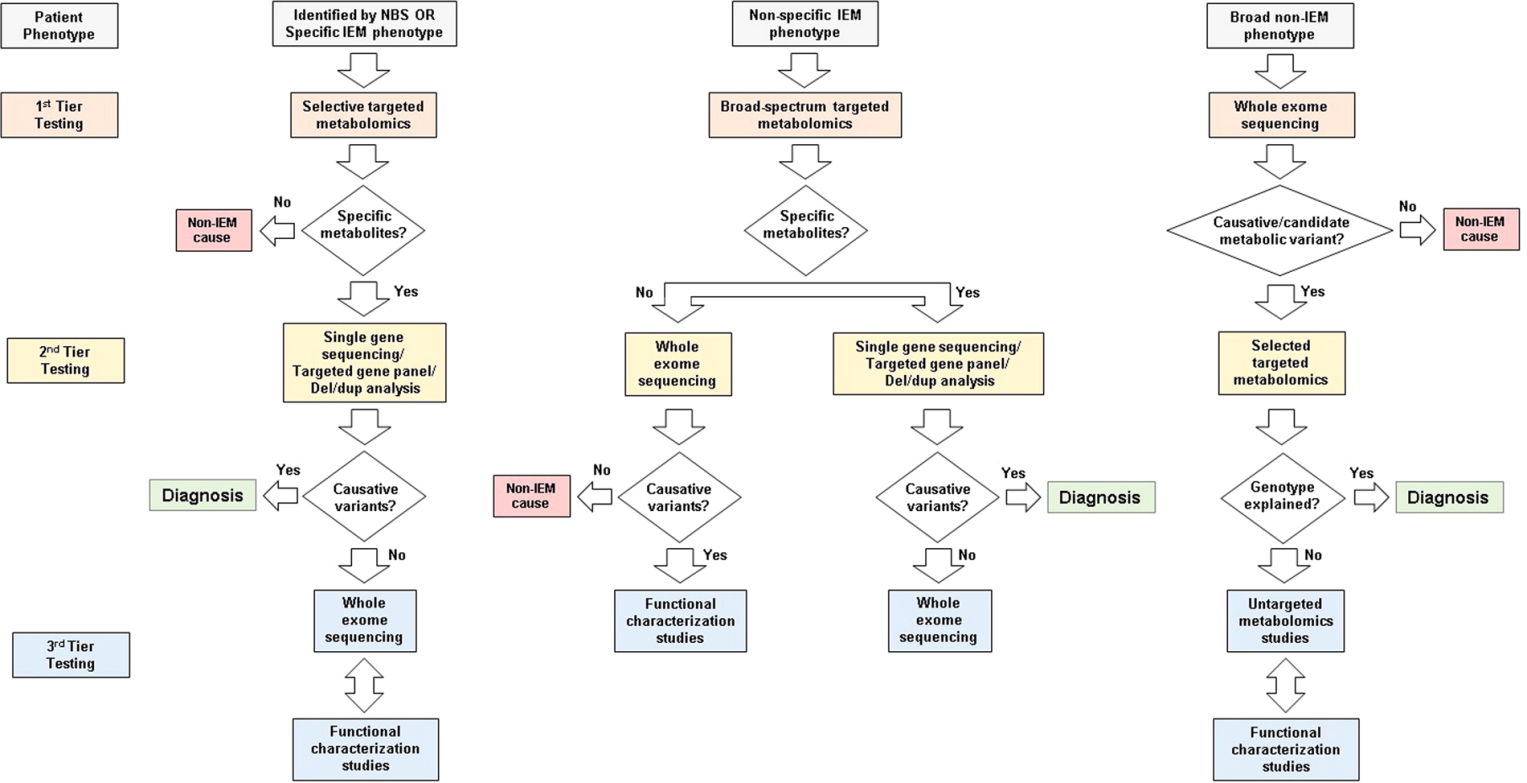
Investigational algorithm for IEM using metabolomics testing. Individuals identified through NBS programs or those with specific IEM features are diagnosed by selected targeted assays and confirmed through gene sequencing. In some cases, WES complemented by functional studies, such as enzyme activity measurement, may be required. Individuals with non-specific IEM phenotypes investigated through broad-spectrum targeted metabolomics prior to further investigation through gene panels. In some cases, WES may be warranted with complementary functional studies. Individuals with broad, non-specific (non-IEM), neurometabolic phenotypes investigated by WES as a front-line investigation. It is possible that such patients may benefit from GUM analysis when VUS are discovered by WES, requiring validation. In addition to metabolomics testing, functional characterization studies (such as enzyme activity measurement or flux studies) should be utilized, where appropriate^31^.

Considering the phenotypic variation of IEMs, and the changing landscape of contemporary tools in clinical use, a proposed investigative approach could be considered. Based on a recent model by Wanders *et al*., Fig. 4 describes an approach for the laboratory investigation of three types of IEM presentation, the most challenging of which is patients with non-specific clinical findings with no immediate indication of an IEM^31^. For such individuals, WES is potentially the most effective front-line investigation^32^. A recent meta-analysis has also endorsed the use of WES as a front-line investigation for the evaluation of unexplained neurodevelopmental disorders^33^. As WES analysis becomes more widespread, the number of equivocal findings, requiring functional validation, are also increasing. As a functional validation tool, targeted metabolomic analysis is limited by its scope of application. This is one potential area where GUM may be useful as a biomarker discovery tool. Based on our study data, the interpretation of metabolic signatures in patients harboring candidate or potentially causative variants represents the most appropriate use of GUM at present. As such, GUM analysis should be reserved for a limited number of investigations where genomics and metabolics data are interpreted together in an integrated “functional genomics” approach. This likely also represents the most cost-effective approach, since the costs associated with GUM analysis remain considerable compared with targeted analyses. From a test order utilization perspective, such investigations should ideally be considered following exhaustive unavailing targeted metabolomic analyses.

Several potential limitations were apparent in this study. Inherent variability in metabolic flux is a challenge for effective sample collection for any type of metabolic assessment. The use of isolated samples effectively generates a “snapshot” of the metabolome, which may not necessarily accurately reflect the true disease state. Additionally, all types of targeted assays were not represented for every patient, rather only those available for comparison in paired samples. This limited the number of metabolites available for direct comparison. Metabolite comparison was also limited by the inclusion of non-quantitative targeted assays, and non-standardized local reference interval data. For the discovery study, only a small number of patients of each clinical phenotype were represented, notwithstanding the fact that these are rare disorders. Additionally, over a third of patients did not have any molecular work-up, limiting the interpretation of metabolomic findings.

In conclusion, GUM undoubtedly shows promise for the investigation of IEMs, notwithstanding the analytical challenges associated with this approach. However, untargeted analysis is not yet ready to completely replace traditional targeted assays for first-line screening for IEM. Rather, the role of global untargeted metabolomics analysis is for discovery and validation of VUS in candidate genes, where it offers value in a multi-omics approach in combination with targeted metabolomic and molecular data as a functional genomics tool.

## Methods

All methods were carried out in accordance with relevant guidelines and regulations.

### Study design and cohort description

In total, 278 patients were included in which GUM and TM studies were conducted between March 2015 and August 2018. Patient records were selected in which GUM and TM data were generated from the same sample (sample pairing). This final group of study subjects (n = 226) was separated into 2 cohorts for data comparison: the diagnostic cohort (n = 87), which comprised of patients with a confirmed diagnosis of an IEM (n = 67) or genetic (n = 20) disorder for evaluation in the *clinical utility study*; and the investigative cohort (n = 139), comprised of patients under investigation in whom a diagnosis was not established for evaluation in the *discovery study*. A summary of the study design is provided in Fig. 1. This protocol was approved by the Boston Children’s Hospital Institutional Review Board (IRB-P00022603).

### Sample collection

In all subjects, plasma, urine or CSF was collected according to standard protocols. Blood collected into lithium heparin containers was transported to the laboratory at room temperature and centrifuged within 1 hour of collection at 5,000 rpm using a Sorvall LC-80 bench-top centrifuge (Thermo-Fisher Scientific, Waltham, MA). Plasma was aliquoted and stored at −20 °C until analysis. Urine and CSF samples were collected into plain containers without preservative, transported to the laboratory at room temperature and aliquoted and stored at −20 °C until analysis. In all cases, metabolomics analysis was performed within 7 days of sample collection.

### Molecular testing

All patients provided written consent and received appropriate pre and post-test genetic counseling. The type of molecular assay was based on the clinical indication following a stepwise-approach to reach a molecular diagnosis. Molecular testing ranged from single gene sequencing to WES and chromosomal microarray (CMA), as per the clinical indication and diagnostic guidelines^14^. Genetic testing was performed at external CLIA certified commercial laboratories.

### Metabolomics studies

A description of TM methodologies is provided in the supplementary materials (Table S1). TM analyses were performed at the Department of Laboratory Medicine, BCH, unless otherwise stated. GUM analysis was performed using the Global Metabolomic Assisted Pathway Screen (Global MAPS) assay (Baylor Genetics, Houston, TX). A full description of the methods incorporated in the global MAPS assay and their analytical performance characteristics has been previously described^11^. For targeted metabolomics data, metabolite concentrations were compared with standard reference intervals, where available. Untargeted metabolomics data was compared by comparing z-scores. In short, *z*-scores were calculated by comparing log transformed median scaled values for a given metabolite to the associated mean and standard deviation for that metabolite in the undiagnosed population^11^.

### Metabolite study

The metabolite study was designed to determine the level of concordance between untargeted and targeted methods in their ability to detect increased or decreased concentrations of metabolites in all 283 samples (164 plasma; 100 urine; 19 CSF) collected from the entire cohort of 226 subjects (Fig. 1). The level of agreement between the two approaches was assessed as follows; for untargeted analysis, metabolites were classified as increased or decreased if z-scores were >2.0 or <2.0, respectively; for targeted analysis using quantitative assays, metabolites were classified as increased or decreased if values were greater than, or less than, the upper or lower limit of the reference intervals, respectively; for targeted analysis using qualitative assays, metabolite classification was based on standard clinical biochemical data interpretation. For a small number of selected metabolites where a sufficiently large number of quantitative data points were available for comparison, the correlation of the two approaches as a function of metabolite concentration was evaluated. For untargeted metabolomics, determination of the z-score has been previously described^22^. For targeted metabolomics, quantitative raw data was transformed into z-score equivalents by subjection to log transformation followed by conversion to z-scores based on population reference interval data. Untargeted data (z-scores) were compared with targeted data (log z-score) by Deming Regression. In cases where log transformation of data was not possible, targeted data was included as absolute concentrations.

### Clinical utility study

The clinical utility study was designed to examine GUM as a potential first-line screening tool for IEM and/or genetic disorders. Metabolite data generated from 115 samples (66 plasma; 42 urine; 7 CSF) collected from 87 patients were interrogated for the presence of diagnostic metabolites. For TM analysis using quantitative assays, metabolites were classified as increased or decreased if values were greater or less than the upper or lower limit of the reference intervals, respectively. For TM analysis using qualitative assays, metabolite classification was based on standard data interpretation. For GUM analysis, metabolites were classified as increased or decreased if *z*-scores were >2.0 or <2.0, respectively. To assess the utility of GUM as a first-line screening tool, the ability to detect diagnostic metabolites (defined as metabolites providing diagnostic information that would either result in a diagnosis, or inform further testing) was examined against traditional TM approaches. The clinical sensitivity was determined for assessed metabolites based on the number of true positives (TP) and false negatives (FN) defined as follows; TP: Metabolite detected by both GUM and TM; FN: Metabolite not detected using GUM, but detected using TM.

### Discovery study

This study was designed to examine GUM as a discovery tool for detecting metabolites that are either associated with a defined clinical phenotype, provide functional data to inform the genotype, and/or add diagnostic utility beyond that achieved through conventional TM investigations^15^. A total of 177 samples (100 plasma, 65 urine, 12 CSF) obtained from 139 patients were included in the discovery study. Data from TM and GUM analyses were interrogated for the presence of metabolites that may explain the patient phenotype or provide correlation with molecular data.

## Supplementary information

Supplementary information.

Supplementary Table S1

Supplementary Table S2

Supplementary Table S3
