# Author Correction: Clinical Validation of Targeted and Untargeted Metabolomics Testing for Genetic Disorders: A 3 Year Comparative Study

**DOI:** 10.1038/s41598-020-68532-y

**Published:** 2020-07-07

**Authors:** Naif A. M. Almontashiri, Li Zha, Kim Young, Terence Law, Mark D. Kellogg, Olaf A. Bodamer, Roy W. A. Peake

**Affiliations:** 1 Department of Laboratory Medicine, Boston Children’s Hospital, Harvard Medical School, Boston, MA USA; 2 Faculty of Applied Medical Sciences and the Center for Genetics and Inherited Disorders, Taibah University, Almadinah Almunwarah, Saudi Arabia; 3 Division of Genetics and Genomics, Boston Children’s Hospital, Harvard Medical School, Boston, MA USA; 4 Broad Institute of Harvard University and MIT, Cambridge, MA USA

Correction to: *Scientific Reports* https://doi.org/10.1038/s41598-020-66401-2, Published online: 10 June 2020

The Acknowledgements section in this Article is incomplete.

“We would like to thank our colleagues in the Department of Laboratory Medicine and the Division of Genetics and Genomics, Boston Children’s Hospital, for their kind support with this project.”

should read:

“The authors extend their appreciation to the Deputyship for Research & Innovation, Ministry of Education in Saudi Arabia, for funding this research work through the project number 821. We would also like to thank our colleagues in the Department of Laboratory Medicine and the Division of Genetics and Genomics, Boston Children’s Hospital, for their kind support with this project.”

