# Supplementary material for: Clinical Validation of Targeted and Untargeted Metabolomics Testing for Genetic Disorders: A 3 Year Comparative Study
Source: Sci Rep. 2020 Jun 10;10:9382. doi: 10.1038/s41598-020-66401-2 (PMC7287104; doi:10.1038/s41598-020-66401-2)
Supplement: Supplementary file 1 — Supplementary information. [file 41598_2020_66401_MOESM1_ESM.docx]

Clinical Validation of Targeted and Untargeted Metabolomics Testing for Genetic Disorders: A 3 Year Comparative Study: Supplemental Materials and Data Set.

Naif AM Almontashiri, PhD^1,2^, Li Zha, PhD^1^, Kim Young, MSc^1^, Terence Law, MSc^1^, Mark D Kellogg, PhD^1^, Olaf A Bodamer, MD, PhD^3,4^, Roy WA Peake, PhD^1*^

^1^Department of Laboratory Medicine, Boston Children’s Hospital, Harvard Medical School, Boston, Massachusetts, USA.

^2^Faculty of Applied Medical Sciences and the Center for Genetics and Inherited Disorders, Taibah University, Almadinah Almunwarah, Saudi Arabia.

^3^Division of Genetics and Genomics, Boston Children’s Hospital, Harvard Medical School, Boston, Massachusetts, USA.

^4^Broad Institute of Harvard University and MIT, Cambridge, Massachusetts, USA

*Corresponding Author:

Roy W.A. Peake, PhD, FACMG

Tel: 617-355-4182

Email: [Roy.Peake@childrens.harvard.edu](mailto:Roy.Peake@childrens.harvard.edu)

**Section S1: Overview of Untargeted Metabolomics Methodology**

A full description of the method is provided in reference 11. In short, small molecules were extracted in 80% methanol solution and subjected to four analyses: Two liquid-chromatography tandem mass spectrometry (LC-MS/MS) analyses in positive ionization mode and two LC-MS/MS analyses in negative ionization mode. All chromatographic separations were completed using an Acquity UPLC (Waters) equipped with either a Waters BEH C18 column or a Waters BEH amide column, depending on the method, followed by analysis with a Q-Exactive high-resolution mass spectrometer (Thermo-Finnigan). Metabolites were identified with known chemical structure by matching the ions chromatographic retention index, nominal mass, and mass spectral fragmentation signatures, with reference library entries created from authentic standard metabolites under the identical analytical procedure as the experimental samples. Currently, the reference library contains entries for ~ 2500 unique human metabolites. Semi quantitative analysis was achieved by comparing patient samples to a set of invariant anchor specimen included in each batch. Raw spectral intensity values were normalized to the anchor samples, log transformed, and compared to a normal reference population to generate z-score values. Rare compounds are those analytes detected in the patient specimen but only rarely seen in the reference population (< 5% of all patients tested). For urine samples, all data are normalized to creatinine prior to metabolomic analysis.

**Section S2: Overview of Targeted Metabolomics Methodologies**

*Qualitative Urine Organic Acids:*-

Urine organic acids were extracted into ethyl acetate/ether and converted to trimethylsilyl (TMS) derivatives using N,O-Bis(trimethylsilyl)trifluoroacetamide (BSTFA) and 1% trimethylchlorosilane (TMCS) prior to gas chromatography mass spectrometry (GCMS) analysis using a GC 6890N/ MS 5975 system equipped with a DB-1 column (Agilent Technologies, Santa Clara, CA). Data was acquired in scan mode between 50 and 550 *m/z.*

*Urine Acylglycines and Quantitative Urine Organic Acids:-*

Urine acylglycines, urine methylmalonic acid, urine glutaric acid and urine ethylmalonic acid were subjected to acidification followed by solid-phase extraction using CHEM Elut, 300mL unbuffered column resin (Agilent Technologies, Santa Clara, CA). Extracted acylglycines were subjected to trimethylsilyl (TMS) derivativatization using BSTFA + 1% TMCS. Derivatized acylglycines were separated on a DB-1 gas chromatography column followed by chemical ionization MS/MS in MRM mode using a GC-QQQ 7000 GC-MS/MS system equipped with a (Agilent Technologies, Santa Clara, CA). Data was acquired in MRM mode. Serum methylmalonic, glutaric, 3-hydroxyglutaric and 2-methylcitric acids were measured by stable isotope dilution GC-MS.

*Plasma Acylcarnitines:-*

Plasma acylcarnitines were esterified by butylation using butanol-HCl followed by extraction with acetonitrile and flow-injection MS/MS using a Shimadzu prominence LC20-AD HPLC system (Shimadzu Corporation, Kyoto, Japan) coupled to an API 5000 tandem mass spectrometer (ABSciex, Concord, ON). Data was acquired in both MRM and parent ion scan mode (*m/z* = 85 Da).

*Plasma Free and Total Carnitine:-*

Plasma free and total carnitines were measured by stable isotope dilution LC-MS/MS using a deuterated internal standard (carnitine-^2^H_9_). Samples were de-esterified by alkaline hydrolysis using NaOH followed by neutralization of free carnitines by addition of HCl and subsequently separated using a Hypersil GOLD analytical column (Thermo-Scientific, Waltham, MA) on a Shimadzu prominence LC20-AD HPLC system (Shimadzu Corporation, Kyoto, Japan) coupled to an API 5000 tandem mass spectrometer (ABSciex, Concord, ON). Data was acquired in MRM mode.

*Amino Acids:-*

Amino acid analysis was performed in plasma/urine or CSF using an Acquity ultraperformance liquid chromatography (UPLC) system with integrated TUV detector and MassTrak AAA Solutions Kit (Waters Corporation, Milford, MA). The MassTrak kit utilizes pre-column derivatization of amino acids with a 6-aminoquinolyl-N-hydroxysuccinimdyl carbamate tag (AccQTag) followed by reversed-phase UPLC on a C18 column (1.7 μm; 2.1 × 150 mm) and UV detection at 260 nm.

*Other Targeted Methods:*

Plasma lactic acid measurement was performed using a lactate oxidase colorimetric assay (Roche Cobas c501). Plasma Myoinositol was measured using reversed-phase HPLC with UV detection. Serum very long chain fatty acids (VLCFAs) were converted to pentafluorobenzyl bromide fatty acid esters followed by capillary GCMS using a SP-2560 column. Serum and urine guanidinoacetate (GUAA) was measured by stable isotope dilution GCMS using 15N, 13C2 GUAA as an internal standard. GAA was derivatized with hexafluoroacetylacetone and pentafluorobenzyl bromide followed by GC using a methylsilicone capillary column. Mass spectrometry was performed by chemical ionization and detection of GUAA in SIM mode.

**Section S3: Metabolite comparison study**

For TM, quantitative data was transformed into z-score equivalents by log transformation followed by conversion to z-scores based on population reference interval data. Where log transformation was not possible, TM data was compared as absolute concentrations. Quantitative data was compared by Deming regression.

**Organic acids**

Comparison of targeted and untargeted metabolomics for methylmalonic acid (MMA) in plasma and urine samples (n = 15) showed concordance in 64% of samples overall, with 100% concordance for urine samples. In plasma, there were 5 samples (**Table S3: F51, F79; Table S4:F124, F180 and F186**) in which mild elevations were detected using TM analyses, but not GUM. Concordance for 2-methylcitric acid (n = 11) and ethylmalonic acid (n = 7) was 100%. In addition, correlation with concentration was observed for several metabolites across wide concentration ranges (**supplementary materials; Figure S1a**). Glutaric acid, compared in plasma (n = 1) and urine (n = 3), showed 75% concordance, with only one patient sample (**Table S4; F131**) showing a mild elevation in urine at 18 mg/g Cr (reference interval: < 15) using TM analysis, not observed with GUM. 3-Hydroxyglutaric acid, a highly specific marker for glutaric aciduria type I (GA I), exhibited 50% concordance. There was discordance noted for several additional compounds, most notably homogentisic acid (**Table S3; F11**). Concordance of 3-hydroxybutyric acid was favorable between both approaches, with most discrepant cases being attributed to mildly elevated cases. Perhaps not surprisingly, acetoacetic acid concordance was not as favorable, with GUM analysis failing to detect any cases (n = 7). Similarly, concordance of lactic and pyruvic acid levels exhibited only minimal concordance at 53% and 13% respectively, likely reflecting the challenges associated with compromised pre-analytical processing and sample stability of these metabolites.

**Amino acids**

Comparative data was obtained for 22 amino acids. In the majority of cases, non-concordance was due to mildly elevated or decreased metabolite concentrations detected by TM analysis, not detected using GUM analysis. Most data was obtained for glycine (n = 22), where concordance was 45% across all sample types. Notably, two specimens with moderately increased plasma glycine levels at 396 and 400 mcmol/L (reference interval: 150 – 305) using targeted analysis exhibited normal z-scores (< 2.0) using GUM. Unsurprisingly, unstable amino acids, such as homocystine, cystine and cystathionine had unsatisfactory levels of concordance, albeit with low sample numbers represented. Two amino acids, argininosuccinic acid and s-sulfocysteine, pathognomonic for argininosuccinic acid lyase and sulfite oxidase deficiencies respectively, were detected in two plasma samples by TM analysis, not observed with GUM analysis.

**Carnitine and acylcarnitines**

Plasma free carnitine levels (n = 22) exhibited concordance of 50% However, all cases of non-concordance involved samples where free carnitine levels were either mildly decreased (range: 17 – 25 mcmol/L), or mildly increased (range: 63 - 69 mcmol/L), using TM analysis, not detected using GUM analysis (**supplementary materials; Figure S1c**). For acylcarnitines, the majority of comparative data was obtained for hexanoylcarnitine (n = 10), where excellent concordance (90%) was achieved between approaches. Similarly, propionylcarnitine concordance was 78% (n = 9). Concordance for glutarylcarnitine was 50%; unsurprisingly, concordance was greatest for confirmed cases of glutaric aciduria, where levels were significantly elevated.

**Other notable metabolites**

Guanidinoacetate, a glycocyamine used for the investigation of creatine deficiency syndromes, exhibited relatively poor concordance between methods with only 20% agreement (n = 5). Similarly, agreement for phytanic and pristanic acids, measured for the investigation of peroxisomal dysfunction, was unsatisfactory in three patients with alpha-methylacyl-CoA racemase deficiency. In contrast, concordance for galactitol, a sugar alcohol primarily used for monitoring patients with galactosemia, showed 80% concordance (n = 5) between approaches.

**Figure S1a: Comparison of quantitative data for Propionyl-CoA metabolites**

Deming regression analysis comparing untargeted (GUM) with targeted (TM) metabolomics analysis of metabolites of propionyl-CoA. Deming, propionylcarnitine: z(GUM) = 3.93 logz(TM) - 0.17; methylmalonate: z(GUM) = 2.45logz(TM) + 1.07; 2-methylcitrate: z(GUM) = 0.80 logz(TM) + 1.3.


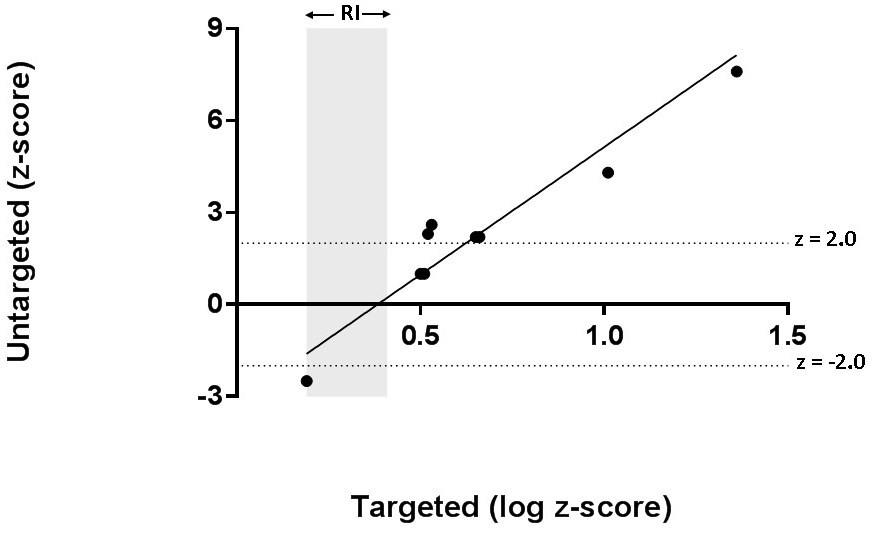


**Figure S1b: Comparison of quantitative data for glycine**

Regression analysis comparing untargeted (GUM) with targeted (TM) metabolomics analysis of glycine. Grey box represents the plasma reference interval (RI) for targeted analysis. Dashed lines represent the upper and lower cut-off values for untargeted analysis. Deming, z (GUM) = 8.327 logz(TM) – 3.2.


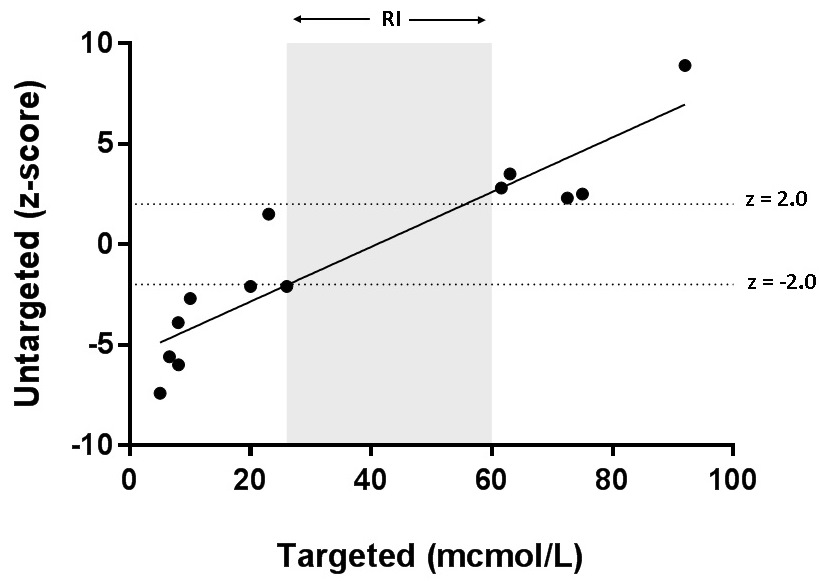


**Figure S1c: Comparison of quantitative data for carnitine**

Regression analysis comparing untargeted (GUM) with targeted (TM) metabolomics analysis of plasma carnitine. Grey box represents the reference interval (RI) in plasma using targeted analysis. Dashed lines represent the upper and lower cut-off values for untargeted analysis. Deming, z (GUM) = 0.14 (TM) – 5.6.


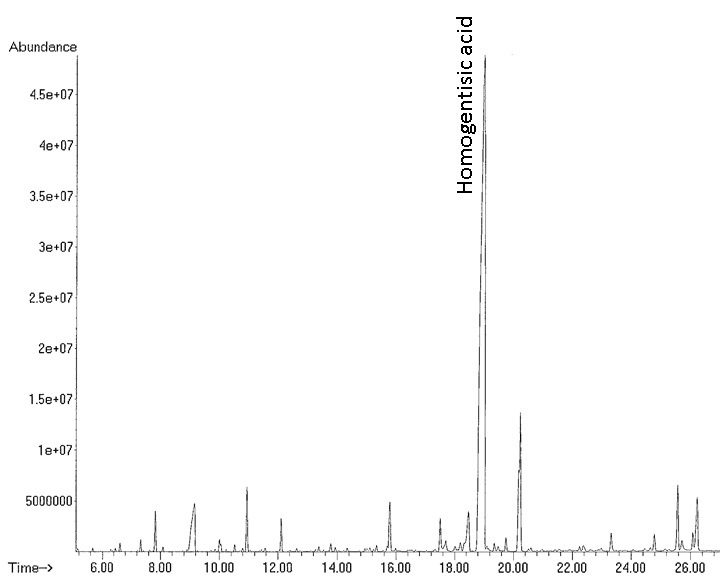


**Figure S2a: Urine Organic Acid Chromatogram: Patient F11-1**

Urine organic acids were extracted into ethyl acetate/ether and converted to trimethylsilyl (TMS) derivatives prior to analysis using a GC 6890N/ MS 5975 system equipped with a DB-1 column (Agilent). A major peak corresponding to homogentisic acid at approximately 18.9 minutes is highlighted.


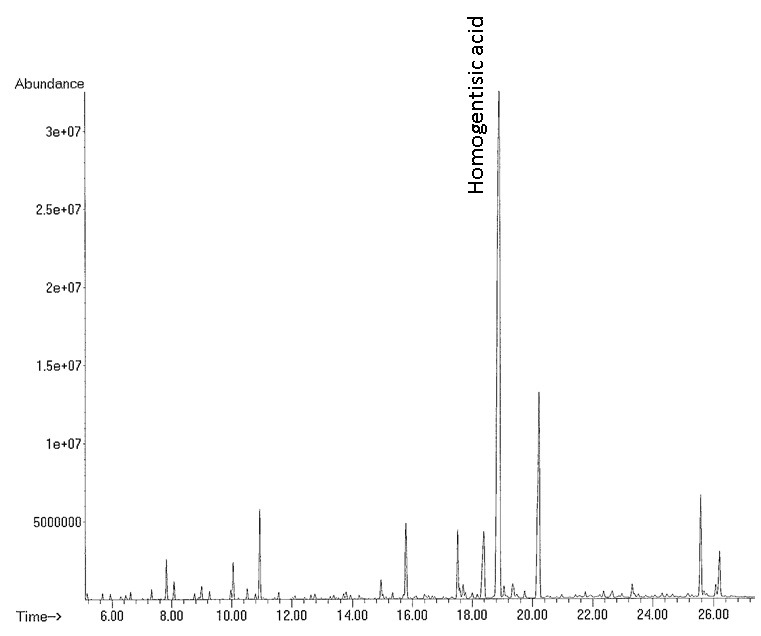


**Figure S2b: Urine Organic Acid Chromatogram: Patient F11-2**

Urine organic acids were extracted into ethyl acetate/ether and converted to trimethylsilyl (TMS) derivatives prior to analysis using a GC 6890N/ MS 5975 system equipped with a DB-1 column (Agilent). A major peak corresponding to homogentisic acid at approximately 18.9 minutes is highlighted.


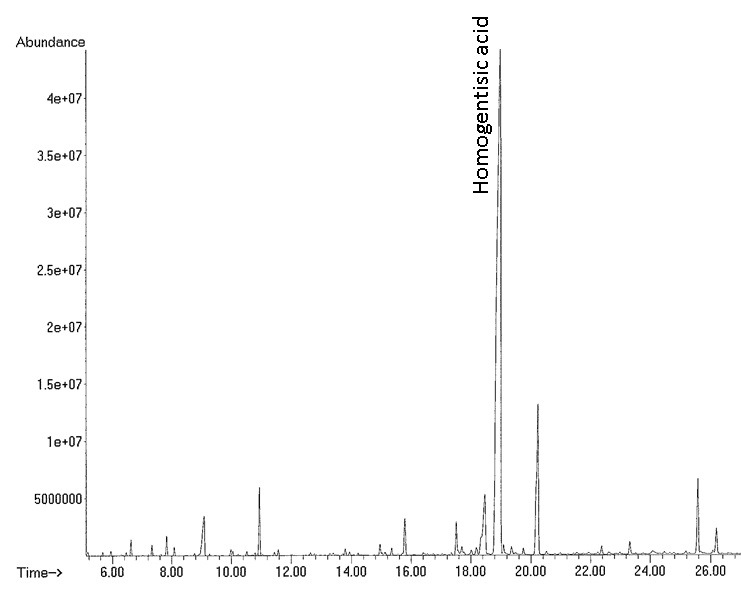


**Figure S2c: Urine Organic Acid Chromatogram: Patient F11-3**

Urine organic acids were extracted into ethyl acetate/ether and converted to trimethylsilyl (TMS) derivatives prior to analysis using a GC 6890N/ MS 5975 system equipped with a DB-1 column (Agilent). A major peak corresponding to homogentisic acid at approximately 18.9 minutes is highlighted.


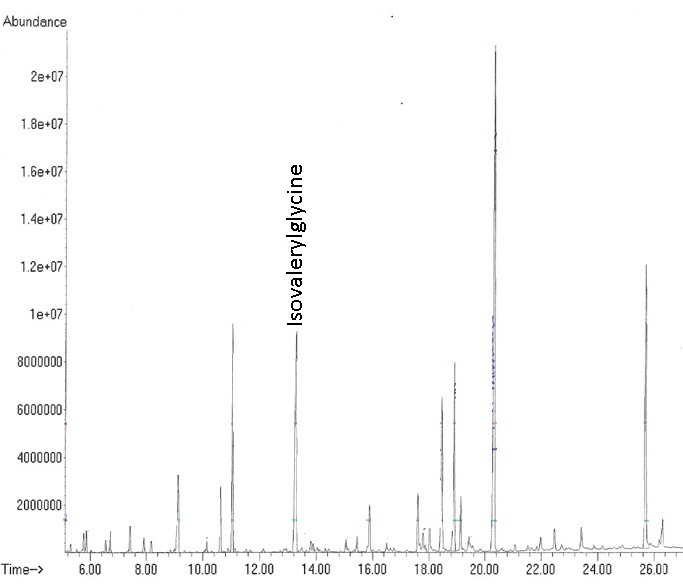


**Figure S3: Urine Organic Acid Chromatogram: Patient F6**

Urine organic acids were extracted into ethyl acetate/ether and converted to trimethylsilyl (TMS) derivatives prior to analysis using a GC 6890N/ MS 5975 system equipped with a DB-1 column (Agilent). A peak corresponding to isovalerylglycine at approximately 13.2 minutes is highlighted.


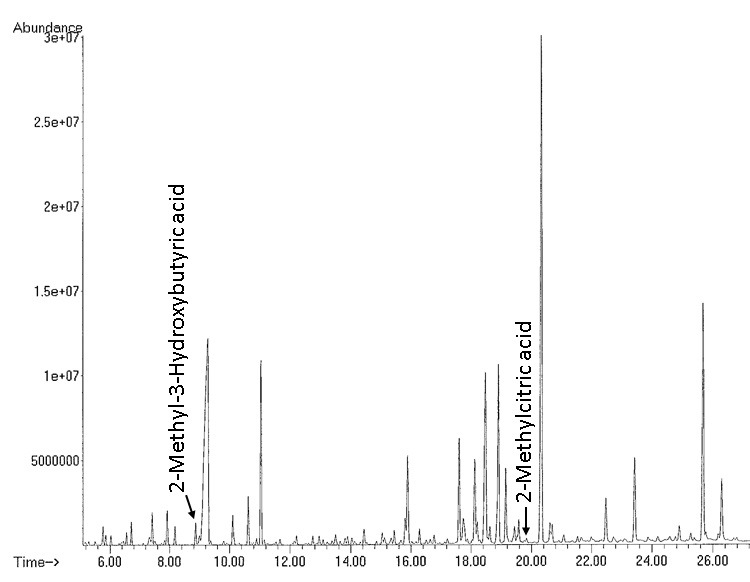


**Figure S4: Urine Organic Acid Chromatogram: Patient F10**

Urine organic acids were extracted into ethyl acetate/ether and converted to trimethylsilyl (TMS) derivatives prior to analysis using a GC 6890N/ MS 5975 system equipped with a DB-1 column (Agilent). Peaks corresponding to 2-methyl-3-hydroxybutyric acid and 2-Methylcitric acid are noted at approximately 8.9 minutes and 19.5 minutes, respectively.


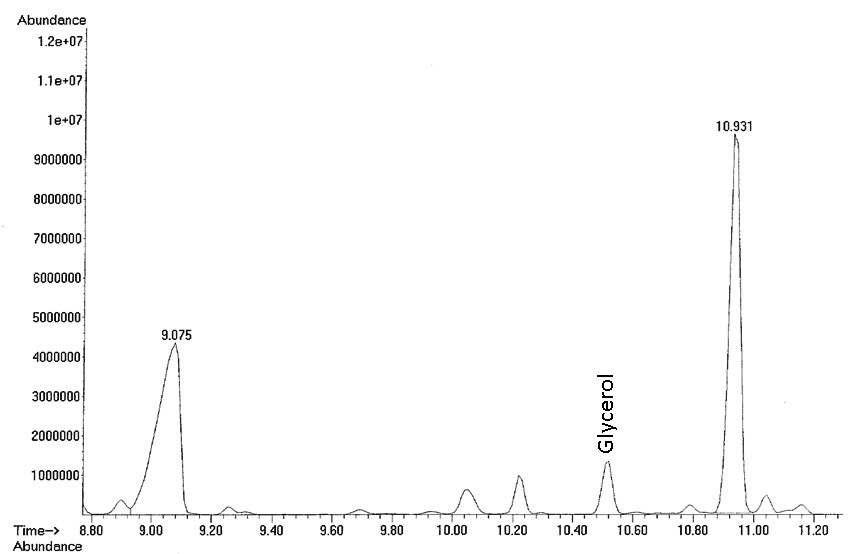


**Figure S5: Urine Organic Acid Chromatogram: Patient F38.**

Urine organic acids were extracted into ethyl acetate/ether and converted to trimethylsilyl (TMS) derivatives prior to analysis using a GC 6890N/ MS 5975 system equipped with a DB-1 column (Agilent). A peak corresponding to glycerol is noted at approximately 10.5 minutes.


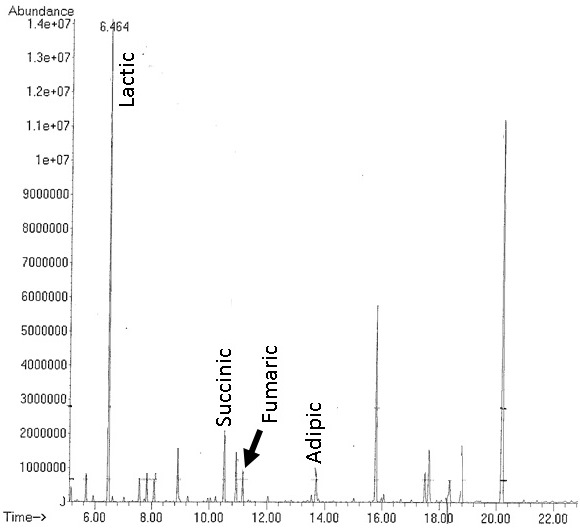


**Figure S6: Urine Organic Acid Chromatogram: Patient F41.**

Urine organic acids were extracted into ethyl acetate/ether and converted to trimethylsilyl (TMS) derivatives prior to analysis using a GC 6890N/ MS 5975 system equipped with a DB-1 column (Agilent). Peaks corresponding to lactate (6.5 min), succinate (10.4 min), fumarate (11.2 min) and adipate (13.7 min) are highlighted.
